# A deep learning method to more accurately recall known lysine acetylation sites

**DOI:** 10.1186/s12859-019-2632-9

**Published:** 2019-01-23

**Authors:** Meiqi Wu, Yingxi Yang, Hui Wang, Yan Xu

**Affiliations:** 1 Department of Information and Computer Science, University of Science and Technology Beijing, Beijing, 100083 China; 2 Institute of Computing Technology, Chinese Academy of Sciences, Beijing, 100190 China; 3 Beijing Key Laboratory for Magneto-photoelectrical Composite and Interface Science, University of Science and Technology Beijing, Beijing, 100083 China

**Keywords:** Lysine acetylation, PTMs, Deep learning

## Abstract

**Background:**

Lysine acetylation in protein is one of the most important post-translational modifications (PTMs). It plays an important role in essential biological processes and is related to various diseases. To obtain a comprehensive understanding of regulatory mechanism of lysine acetylation, the key is to identify lysine acetylation sites. Previously, several shallow machine learning algorithms had been applied to predict lysine modification sites in proteins. However, shallow machine learning has some disadvantages. For instance, it is not as effective as deep learning for processing big data.

**Results:**

In this work, a novel predictor named DeepAcet was developed to predict acetylation sites. Six encoding schemes were adopted, including a one-hot, BLOSUM62 matrix, a composition of K-space amino acid pairs, information gain, physicochemical properties, and a position specific scoring matrix to represent the modified residues. A multilayer perceptron (MLP) was utilized to construct a model to predict lysine acetylation sites in proteins with many different features. We also integrated all features and implemented the feature selection method to select a feature set that contained 2199 features. As a result, the best prediction achieved 84.95% accuracy, 83.45% specificity, 86.44% sensitivity, 0.8540 AUC, and 0.6993 MCC in a 10-fold cross-validation. For an independent test set, the prediction achieved 84.87% accuracy, 83.46% specificity, 86.28% sensitivity, 0.8407 AUC, and 0.6977 MCC.

**Conclusion:**

The predictive performance of our DeepAcet is better than that of other existing methods. DeepAcet can be freely downloaded from https://github.com/Sunmile/DeepAcet.

**Electronic supplementary material:**

The online version of this article (10.1186/s12859-019-2632-9) contains supplementary material, which is available to authorized users.

## Background

Post-translational modifications (PTMs) refer to the chemical modification of a protein after translation. PTMs play a crucial role in regulating many biological functions, such as protein localization in the cell, protein stabilization, and the regulation of enzymatic activity [1]. Studies have shown that 50–90% of the proteins in the human body undergo PTMs, mainly through the splicing of the peptide chain backbone, the addition of new groups to the side chains of specific amino acids, or the chemical modification of existing groups. Acetylation is one of the most important and ubiquitous PTMs in proteins. Protein acetylation is a widespread covalent modification in eukaryotes that occurs by transferring acetyl groups from acetyl coenzyme A (acetyl CoA) to either the α-amino (*N*^*α*^) group of amino-terminal residues or to the ε-amino group (*N*^*ε*^) of internal lysines at specific sites [2]. The lysine acetylation catalyzed by histone acetyltransferases (HATs) or lysine acetyltransferases (KATs) reversibly regulates a large number of biological processes [3]. The function of lysine acetylation in histones to control gene expression by modifying the chromatin structure has been widely studied [4]. Recent studies in proteomics have shown that most acetylation events occur on non-chromatin associated proteins and play an important role in cell signaling and metabolism, protein activities and structure, and sister chromatid polymerization [5–7]. In addition to histone acetylation, non-histone acetylation is also important. Some studies have shown that acetylated non-histones affect the stability of mRNA, intracellular localization, protein-protein interactions, enzyme activity and transcriptional regulation [2, 8, 9]. In addition, most non-histone proteins targeted by acetylation are associated with cancer cell proliferation, tumorigenesis and immune functions [10].

Although a large number of lysine acetylated proteins have been identified, there are still many acetylated proteins that need to be identified. The mechanism of protein acetylation is still largely unknown. The identification of acetylation sites will be an essential step in understanding the molecular mechanisms of protein acetylation. Also, some cancer [11, 12], neurodegenerative disorders [13, 14] and cardiovascular diseases [15, 16] are related to aberrant lysine acetylation. Thus, the identification of acetylation sites can provide a certain guidance for the treatment of some diseases [17]. Kim et al. [18] first developed a method for detecting lysine acetylation sites at the proteomic level by enriching acetylated peptides with lysine acetylated-specific antibodies. Choudhary et al. [19] used high-resolution mass spectrometry to identify 3600 lysine acetylation sites on 1750 proteins. However, the experimental identification of lysine acetylation is very laborious with long periods, for high cost and low throughput. It is necessary to predict the lysine acetylation sites through better approaches.

**Fig. 1:**
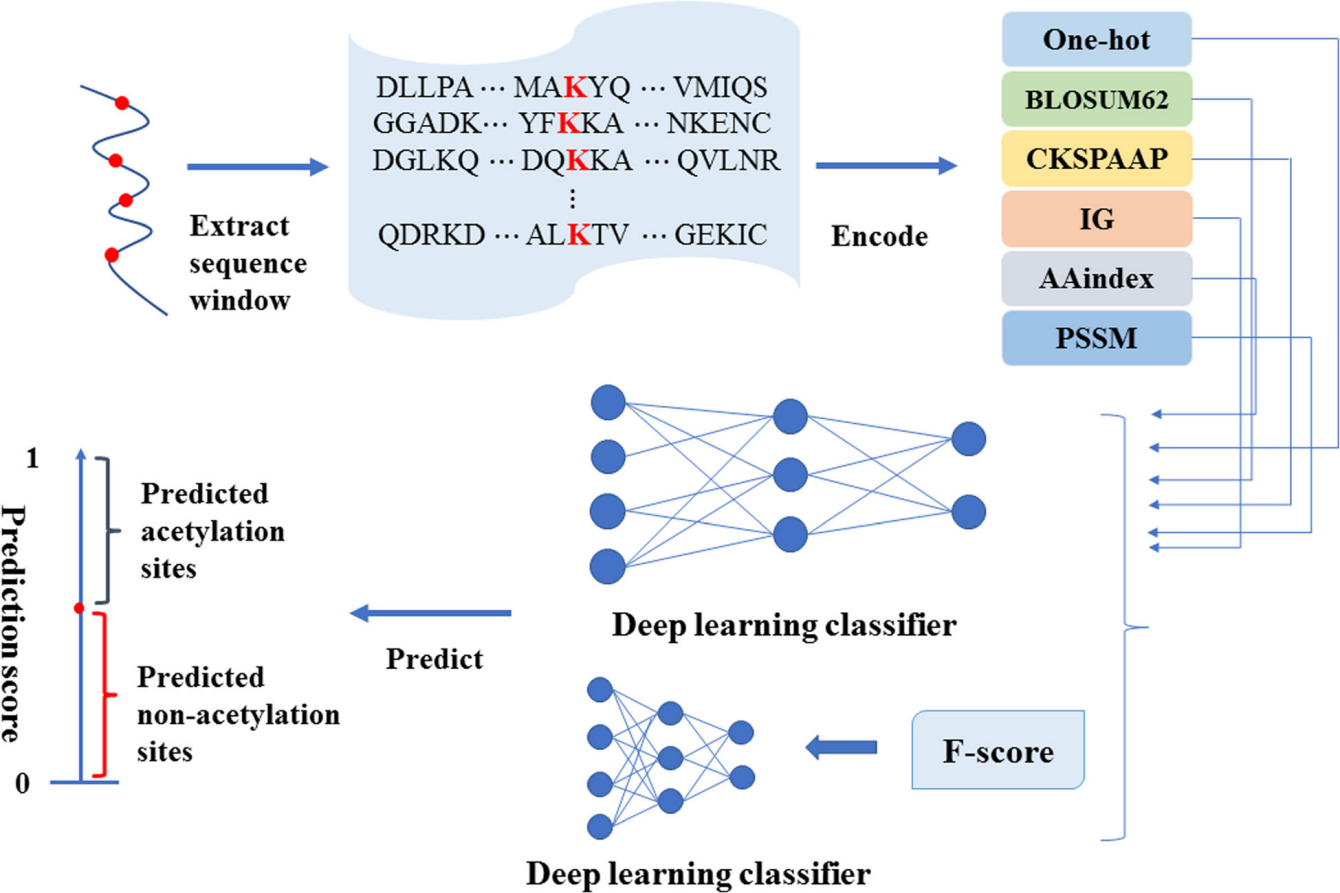
The computational framework of the predictor. Step 1, a peptide of the length of 31 with a center lysine (K) was used to extract sequences from the acetylated proteins. Step 2, six different encoding schemes that are described in Section 2.2 were utilized to encode fragments. Step 3, these six groups of encoded features were used to the train model in two ways. Step 4, the predicted results of the samples

In contrast with time-consuming and expensive experimental methods, computational tools represent an alternative method for studying acetylation. Various machine learning algorithms have been used to predict acetylation sites, such as support vector machine (SVM) [20–23], Bayesian discrimination [24], and logistic regression [25]. These predictors, obtained from shallow machine learning algorithms, have generated good predictions. However, there is still much room for improvement. First, the existing tools generally use machine learning methods. Although NetAcet [26] adopted a neural network, regrettably, the training dataset was very limited during development. With the increase in identified acetylation sites, deep learning has certain advantages for dealing with big data. Second, these methods cannot extract the underlying features of the acetylated protein. To tackle these problems, we proposed a new predictor, DeepAcet, which can extract the high-level features and obtain better predictive results. We adopted two ways to the train models. One way utilized different encoding schemes. The other integrated six types of encoding schemes with an F-score to train the model (Fig. 1).

## Results

### Performance of DeepAcet

**Fig. 2:**
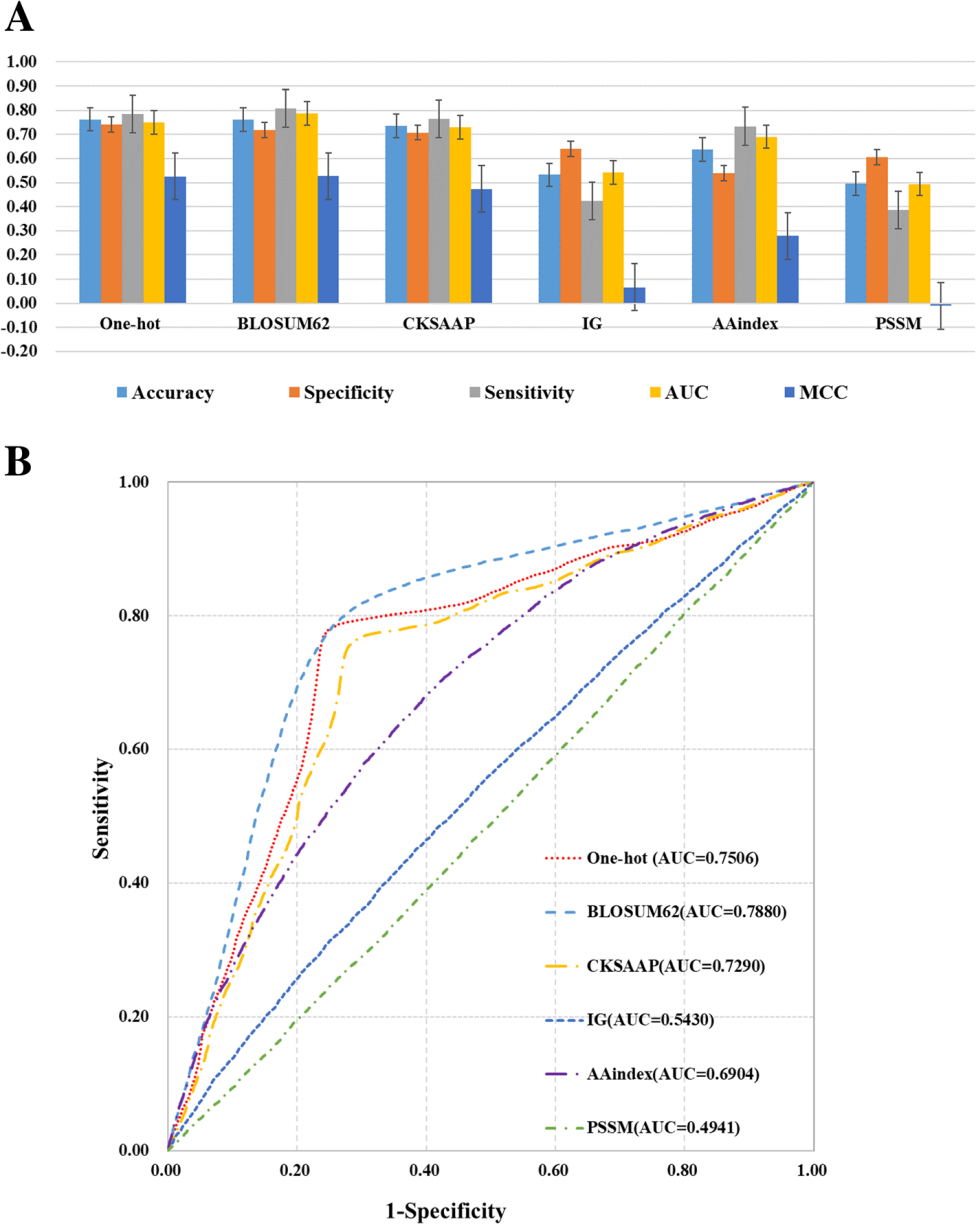
Performance measures for the different features. **a** The Accuracy, Specificity, Sensitivity, AUC values of different features and their error bars. **b** ROC curves and their AUC values for different features

To obtain comprehensive information for the sequences, we chose different encoding schemes which contained sequence location information, amino acid composition information, evolutionary information and physicochemical properties. Different features will have different predictive performance. We first applied a 4-fold cross-validation to test the predictive abilities for the predictors of each encoding scheme. The results showed that different types of features have different contributions to predictive performance (Table 1 , Fig. 2). The BLOSUM62 scheme was the most effective feature for prediction, with an accuracy of 76.23%, specificity of 71.68%, sensitivity of 80.77%, AUC of 0.7880, and MCC of 0.5267. The next most effective schemes were the one-hot, CKSAAP, and AAindex features.

**Table 1:** Performance measures and dimensions for the different features

Feature	Dimension	Accuracy	Specificity	Sensitivity	AUC	MCC
One-hot	651	76.25%	74.00%	78.50%	0.7506	0.5256
BLOSUM62	651	76.23%	71.68%	80.77%	0.7880	0.5267
CKSAAP	2205	73.61%	70.79%	76.44%	0.7290	0.4731
IG	1	53.22%	64.02%	42.43%	0.5430	0.0660
AAindex	434	63.65%	53.92%	73.38%	0.6904	0.2783
PSSM	30	49.50%	60.46%	38.53%	0.4941	−0.0103
Word2vec	31	52.78%	56.89%	48.57%	0.4382	0.1814

From published articles, it is known that a combination of different features makes a model better. Therefore, our next step was to test the predictive performance of combined features. We utilized the CKSAAP encoding scheme and obtained a 2205-dimension featured vector, a 651-dimension featured vector from the one-hot or BLOSUM62, a 434-dimension featured vector from the 14 physicochemical properties from AAindex, a 1-dimension featured vector from IG and a 30-dimension featured vector from the PSSM encoding scheme. The total dimension of features was 3972. We utilized all the features without feature selection as an input to the neural network and K-fold (*k* = 4, 6, 8, 10) cross-validation to evaluate their predictive performance (Additional file 1: Table S1).

**Fig. 3:**
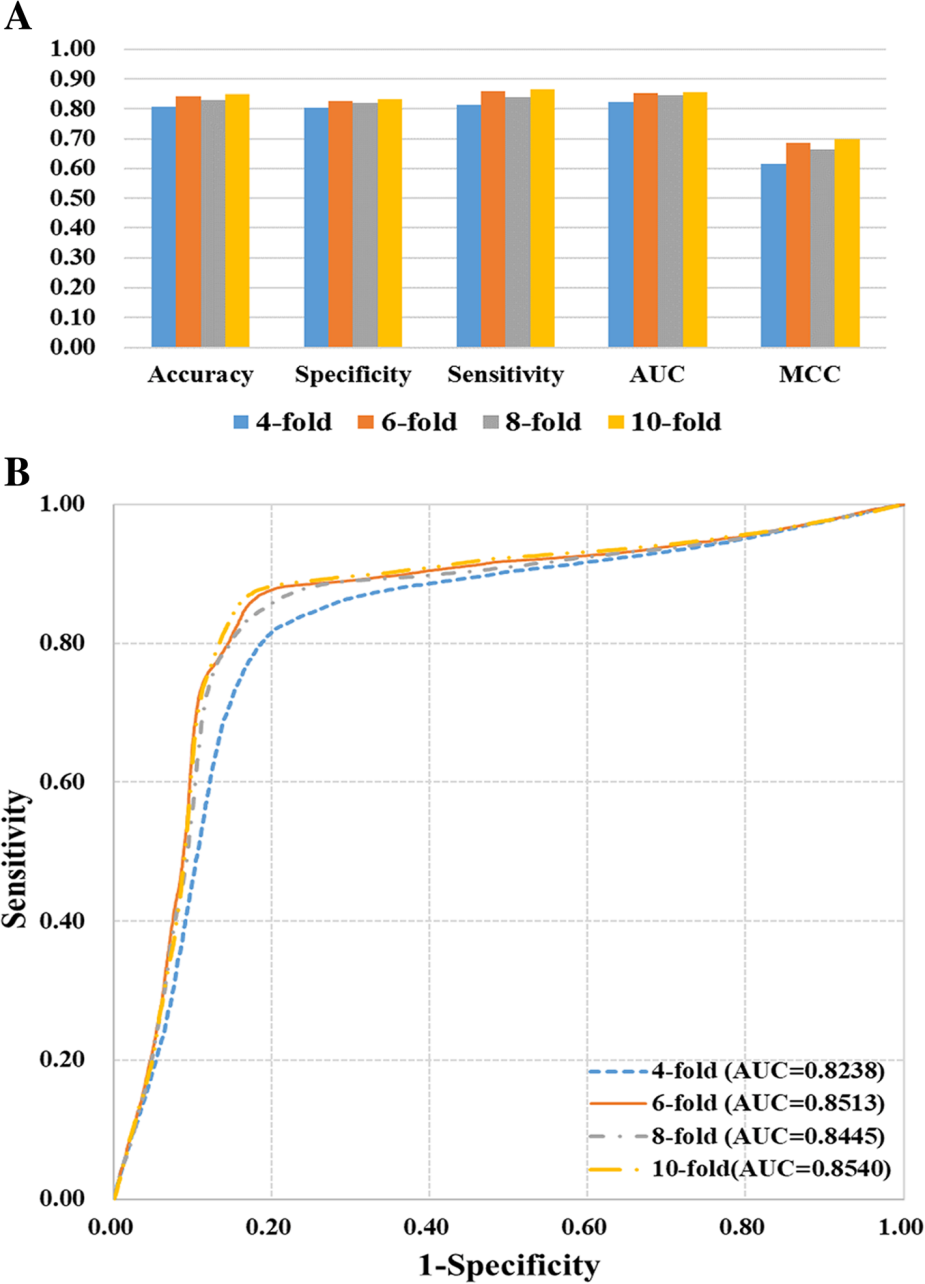
Performance measures of the predictors trained by the optimal features. **a** The Accuracy, Specificity, Sensitivity, AUC values in 4-, 6-, 8-, and 10-fold cross-validation. **b** ROC curves and their AUC values in 4-, 6-, 8-, and 10-fold cross-validation

It is known from these references [27, 28], that some features are redundant and have no contribution to the prediction. Therefore, we calculated the *F*-score for each feature and selected 2199 features with values greater than 0.0001 as the optimal feature set (Additional file 2: Table S2). As expected, the predictive accuracy greatly improved from the selected features (Table 2, Fig. 3). All the accuracy, specificity and sensitivity values were over 80%, with the ACC over 0.8, and the MCC over 0.6. Based on the selected features, the best predictive performance was achieved with 84.95% accuracy, 83.45% specificity, 86.44% sensitivity, 0.8540 AUC, and 0.6993 MCC in a 10-fold cross-validation. Additionally, the ROC curves in 4-, 6-, 8- and 10-fold cross-validation were very close to each other, which illustrated the robustness of the predictor.

**Table 2:** Performance measures for the 4-, 6-, 8-, and 10-fold cross-validations

Cross-validation	Accuracy	Specificity	Sensitivity	AUC	MCC
4	80.79%	80.30%	81.29%	0.8238	0.6159
6	84.28%	82.76%	85.80%	0.8513	0.6858
8	83.12%	82.16%	84.08%	0.8445	0.6625
10	84.95%	83.45%	86.44%	0.8540	0.6993

### Analysis between lysine acetylation and non-acetylation fragments

**Fig. 4:**
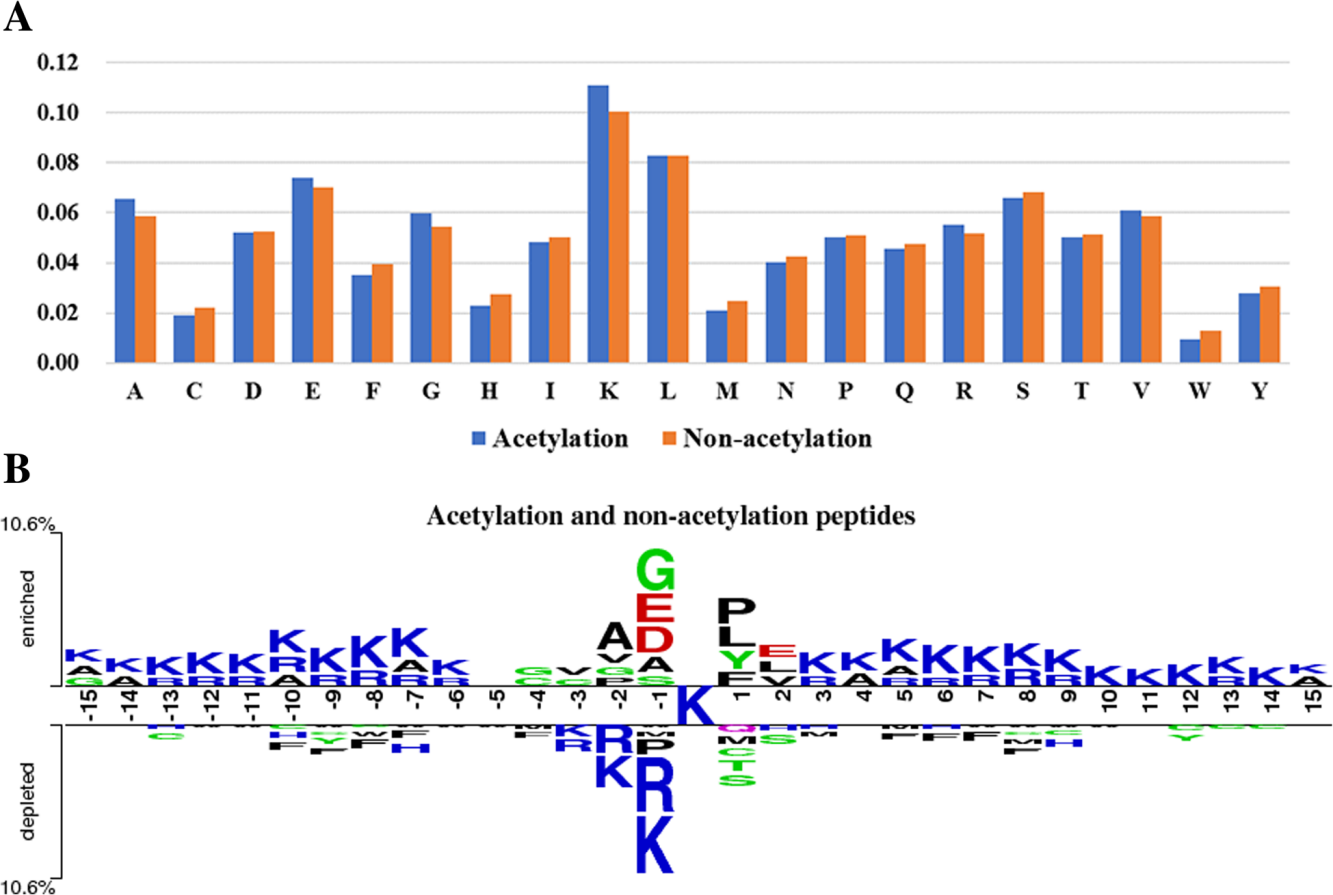
Comparison of between the lysine acetylation fragments and non-acetylation fragments. **a** The percentage of amino acids in the lysine acetylation and non-acetylation fragments. **b** A Two Sample Logo (*p* < 0.0001) of the compositional bias around the lysine acetylation and non-acetylation fragments

We calculated the occurrence composition for various amino acids in the positive and negative datasets to directly observe the differences between lysine acetylated and non-acetylated fragments (Fig. 4a). Also, a Two Sample Logo [29] was utilized to analyze the occurrence of amino acids around lysine acetylation and non-acetylation (Fig. 4b). From Fig. 4a, we can observe that there is certainly a difference in the amino acids between acetylation and non-acetylated fragments. The acetylated fragments contained more alanine (A), glutamic acid (E), glycine (G), lysine (K), arginine (R) and valine (V) than in the non-acetylated fragments. Figure 4b further illustrates that the compositional and positional information of acetylated and non-acetylated fragments have statistically significant differences.

### Optimal features analysis

**Fig. 5:**
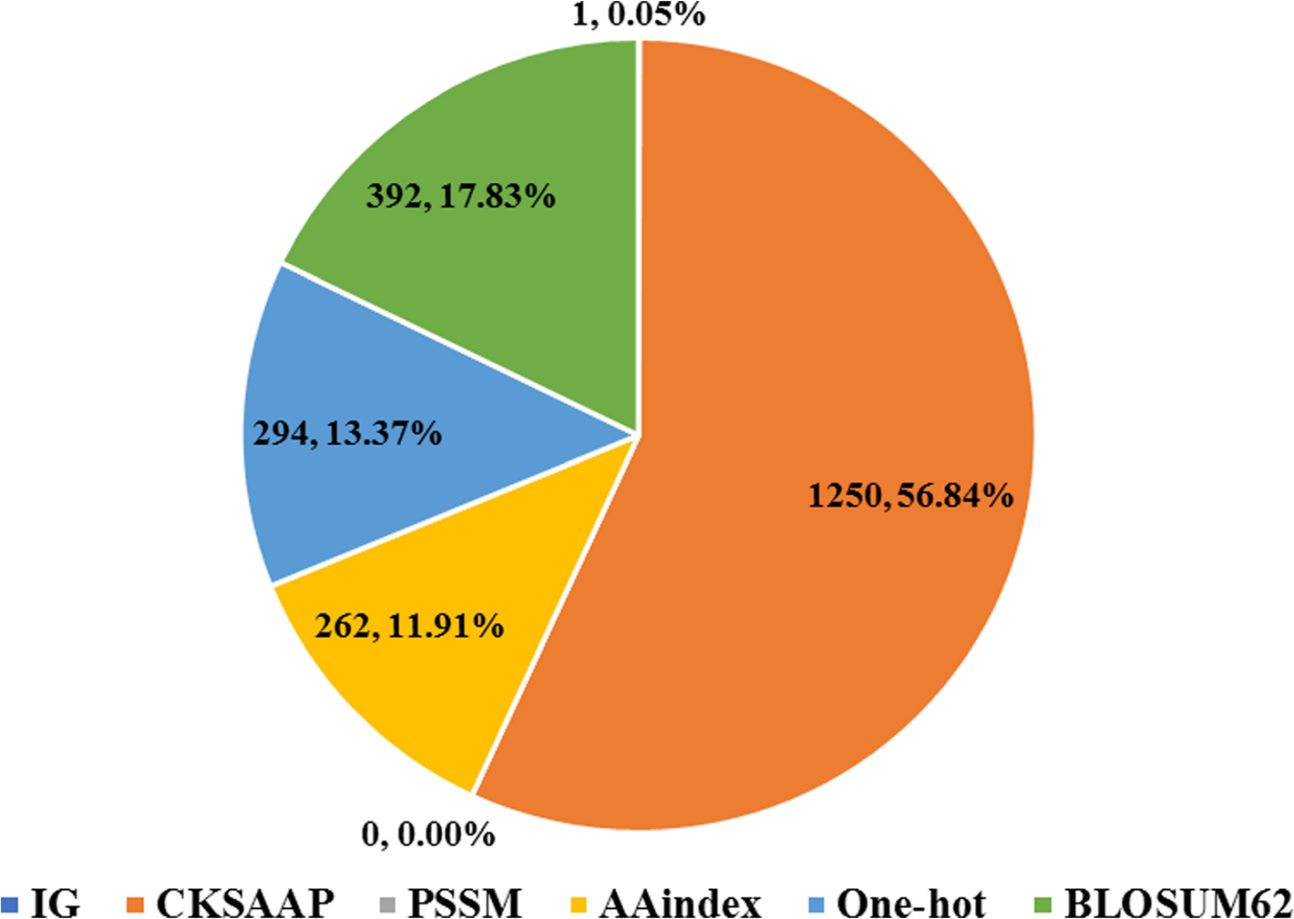
The number of distributions and their percent for each feature. In the 2199 optimal features, 1250 belong to the CKSAAP, 392 to the BLOSUM62, 294 to the one-hot, 262 to the AAindex, 1 to the IG, and 0 to the PSSM

The distribution for each type of feature in the optimal feature set is shown in Fig. 5. In the 2199 optimal features, 1250 belong to the CKSAAP, 392 to the BLOSUM62, 294 to the one-hot, 262 to the AAindex, 1 to the IG, and 0 to the PSSM, suggesting that different features offer different contributions to the classifier. The number of CKSAAP features make up the largest proportion with 56.84%, followed by BLOSUM62 with 17.83%, One-hot with 13.37%, and AAIndex with 11.91%. The sequence encoding scheme CKSAAP utilized different k for the amino acid pair information. BLOSUM62 calculated the similarity of different sequences in the proteins, and AAIndex used the physiochemical properties of the proteins. These optimal features come from different aspects of the proteins, which have different contributions for prediction.

As described above in section 2.2, we selected five different *K* (0, 1, 2, 3, 4) values, respective to each CKSAAP encoding scheme. The total number of features for the optimal feature set with different *K* values is shown in Table 3. It can be seen from the table that these five K values have similar contributions to the optimal feature set.

**Table 3:** Total number of features for the different *K* values

K value	Number
0	253
1	254
2	259
3	242
4	242

### Comparison with other existing methods

**Fig. 6:**
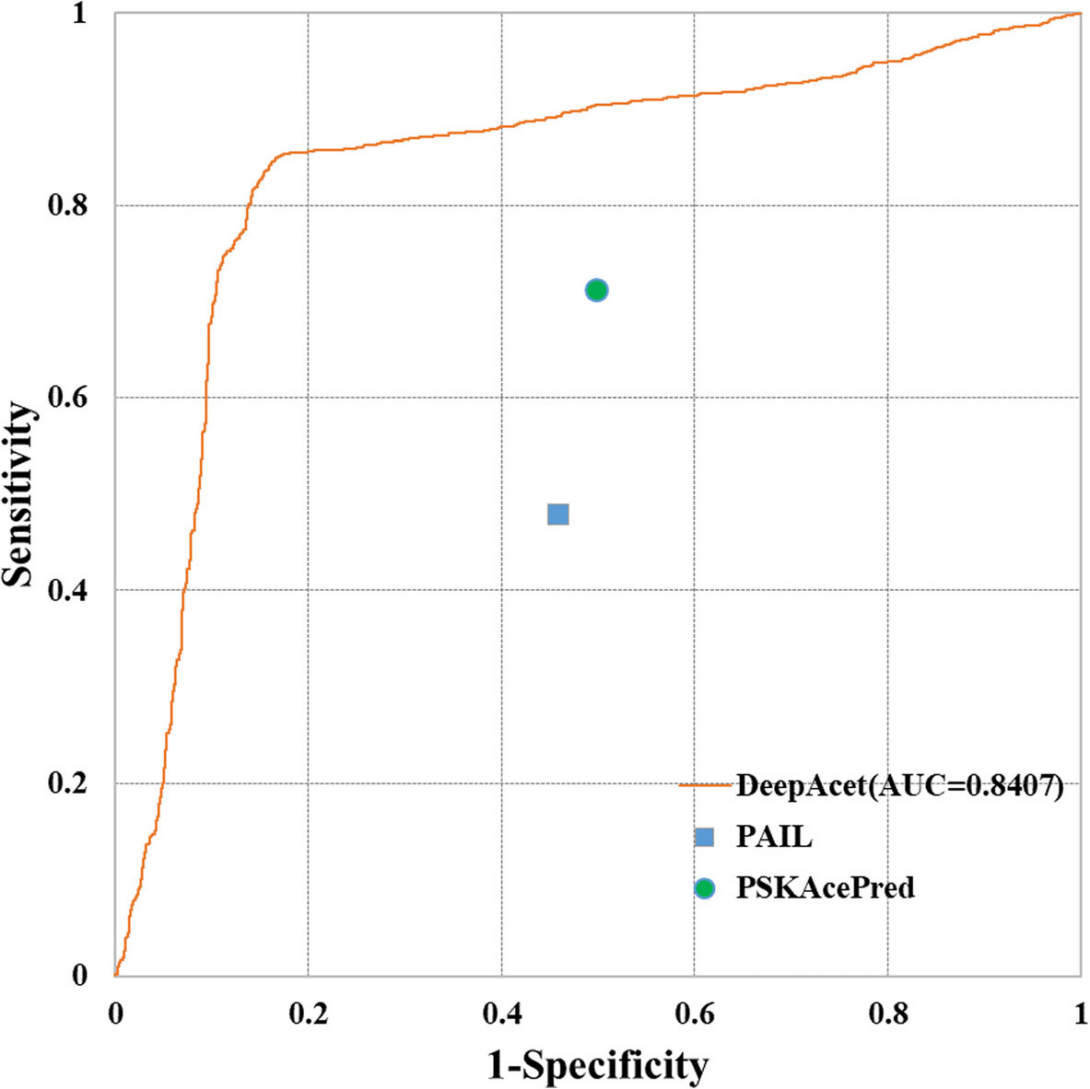
The ROC curve for the independent test set. DeepAcet got the better result than that in PAIL and PSKAcePred

Comparison with different methods should base on same learning dataset. The results will be unfairness if we use different training data. The algorithms will also obtain different results for different feature constructions. However, we couldn’t access the source codes of other existing tools. Another suitable method is to test same independent data which do not been contained in training dataset. In this work, we adopted the later. To demonstrate the performance of our predictor DeepAcet, we further compared our predictor with other existing tools such as PAIL [24], PSKAcePred [23], LAceP [25], N-Ace [20], and BRABSB-PHKA [21], which were trained by shallow machine learning algorithms. We utilized the independent test set described in section 2.1 to test the best performance predictor. The results of the comparison are shown in Table 4 and Fig. 6. However, some prediction tools’ websites were unavailable [20, 21, 25]. Our deep learning predictor DeepAcet had an accuracy of 84.87%, specificity of 83.46%, sensitivity of 86.28%, AUC of 0.8407, and MCC of 0.6977, which were significantly better than the other two predictors.

**Table 4:** Comparision of the performance results with different webserver tools

Prediction method	Algorithms	Accuracy	Specificity	Sensitivity	AUC	MCC
DeepAcet	DL	84.87%	83.46%	86.28%	0.8407	0.6977
PAIL	BDM	51.16%	54.30%	48.04%	–	0.0233
PSKAcePred LAceP N-Ace BRABSB-PHKA	SVM LR SVM SVM	61.01% --- --- ---	50.52% --- --- ---	71.51% --- --- ---	--- --- --- ---	0.2250 --- --- ---

## Discussion

In this work, a satisfactory predictor which could predict unknown acetylation sites, DeepAcet, was obtained by multilayer perceptron from the combination of various encoding schemes. For a long time, researchers have mainly used shallow machine learning algorithms and their methods to predict modified lysine sites. However, in practical application, shallow machine learning is not good for the extraction of high-level features and has poor predictive performance when processing large data. Shallow machine learning uses machine learning algorithms to parse data, learn data features and make decisions or predictions. Deep learning simulates the structure and function of the human brain by identifying the unstructured input of representative data and making accurate decisions. In recent years, deep artificial neural networks have received more and more attention and have been widely applied to image and speech recognition, natural language understanding, and computational biology [30–34]. By propagating data in a deep network, it can effectively extract data features and highly complex functions to improve the classification ability of predictors. Therefore, a deep neural network is used in this work. Deep neural networks can also better handle high-dimensional encoding vectors by training complex multi-layer networks.

The length of input peptides to learning architecture is also one of the hyperparameters. In the prediction of posttranslational modifications, the general range for protein fragments are 21–41. We also tested several lengths such as 21, 23, 25, 27, 29, 33 and 35 on our benchmark data and found that 31 was the best length (Additional file 3: Table S3).

Although we implemented a deep learning framework to build the model and got good results, there is still room for improvement. First, we only considered the composition and location information for the fragments and didn’t consider structural features. Secondly, there is no systematic method to adjust the hyperparameters (e.g., the number of neurons and the number of iterations) of the neural network, which can only be adjusted through the constant experimentation. In the future, we will consider structural information into the features and the new neural network. We could obtain better robustness and accuracy with more experimentally verified acetylation sites. Meanwhile, researchers have found acetylation is associated with diseases [35–37]. We could do some work about the acetylation modification with the disease association.

## Conclusion

Lysine acetylation in protein has become a key post-transcriptional modification in cell regulation [38]. To fully understand the molecular mechanism for the biological processes associated with acetylation, a preliminary and critical step is to identify the acetylated substrates and the corresponding acetylation sites. Therefore, the prediction of acetylation sites through computational methods is desirable and necessary. We built a predictor, DeepAcet, from six features based on a deep learning framework. To get the best predictor, feature selection was utilized to reduce meaningless ones. The predictor achieved an accuracy of 84.95%, specificity of 83.45%, sensitivity of 86.44%, AUC of 0.8540, and MCC of 0.6993 in a 10-fold cross-validation. For the independent test set, the predictive performance achieved an accuracy of 84.87%, a specificity of 83.46%, a sensitivity of 86.28%, AUC of 0.8407, and MCC of 0.6977, results which were significantly superior to those of other predictors. DeepAcet can be freely downloaded from https://github.com/Sunmile/DeepAcet.

## Methods

### Benchmark dataset

We retrieved 29,923 human lysine acetylated sites from the CPLM database (http://cplm.biocuckoo.org/) [39] and their proteins from UniProt (http://www.uniprot.org/). These proteins were truncated with a centered lysine (K) to a fragment length of 31 after many trials. The missing amino acids were filled with the pseudo amino acid “X”. We assigned fragments with the experimental lysine acetylation site into the positive dataset, *S*^+^, and the other fragments into the negative dataset, *S*^−^. In general, if the training dataset had high homology, over-fitting would occur during the training process, which would reduce the generalization ability of the classifier. If more than 30% of the residues in the two comparison fragments were same, only one of them was retained and the other was deleted. After removing the redundant fragments, we obtained 16,107 positive and 57,443 negative fragments. Since the imbalance of a training dataset would cause prediction errors, we randomly selected 16,107 negative fragments from the original dataset, *S*^−^.

Particularly, to evaluate the performance of our prediction model and compare it with other existing tools, we built an independent test set. The independent test set was obtained by randomly selecting one-fifth of the samples from the positive and negative datasets. The remaining samples were used to train the model. Finally, 6442 samples were selected for the independent test set, which contained 3221 positive samples and 3221 negative samples. In the training set, there were 12,886 positive samples and 12,886 negative samples. The detailed statistics of each dataset are shown in Table 5. Detailed information on the training samples and independent test samples are available in Additional file 4: Table S4 and Additional file 5: Table S5, respectively.

**Table 5:** The number of samples for the imbalanced, balanced, training and independent test sets

	Imbalanced dataset	Balanced dataset	Training	Independent test
Positive	16,107	16,107	12,886	3221
Negative	57,443	16,107	12,886	3221

### Feature constructions

All existing operation engines can only handle vectors but not sequence samples [40]. Thus, an important step before training the model was to convert the sequences into numerical vectors that the algorithm could recognize directly. This process is known as feature encoding or feature construction. In this work, six encoding schemes including the basic position, evolutionary information and physicochemical properties were used to construct features. One-hot, Blosum62, Composition of K-space amino acid pairs (CKSAAP), Information gain (IG), AAIndex, and Position-specific scoring matrix (PSSM) are available in the Additional file 6: S6.

### Feature selection

It is necessary to remove redundant features to train the model. Through feature selection, a model can improve its predictive performance with a lower computational cost. An F-score is a simple but effective technique for evaluating the discriminative power of each feature in the feature set [41]. Given the *i* – th feature vector {*p*_*i*1_, *p*_*i*2_, ⋯*p*_*in*_, *n*_*i*1_, *n*_*i*2_, ⋯*n*_*im*_}, the F-score of the *i*–th feature is calculated by

1 $$ F(i)=\frac{{\left({\overline{p}}_i-{\overline{s}}_i\right)}^2+{\left({\overline{n}}_i-{\overline{s}}_i\right)}^2}{\frac{1}{n-1}{\sum}_{k=1}^n{\left({p}_{ik}-{\overline{p}}_i\right)}^2+\frac{1}{m-1}{\sum}_{k=1}^m{\left({n}_{ik}-{\overline{n}}_i\right)}^2} $$

where ${\overline{p}}_i$,${\overline{n}}_i$,${\overline{s}}_i$ are the average of the positive, negative, and whole samples, respectively. *n*, *m* are the number of positive and negative samples, respectively. The larger the *F*-score value, the greater the influence of this feature for predictive performance.

### Operation algorithm

**Fig. 7:**
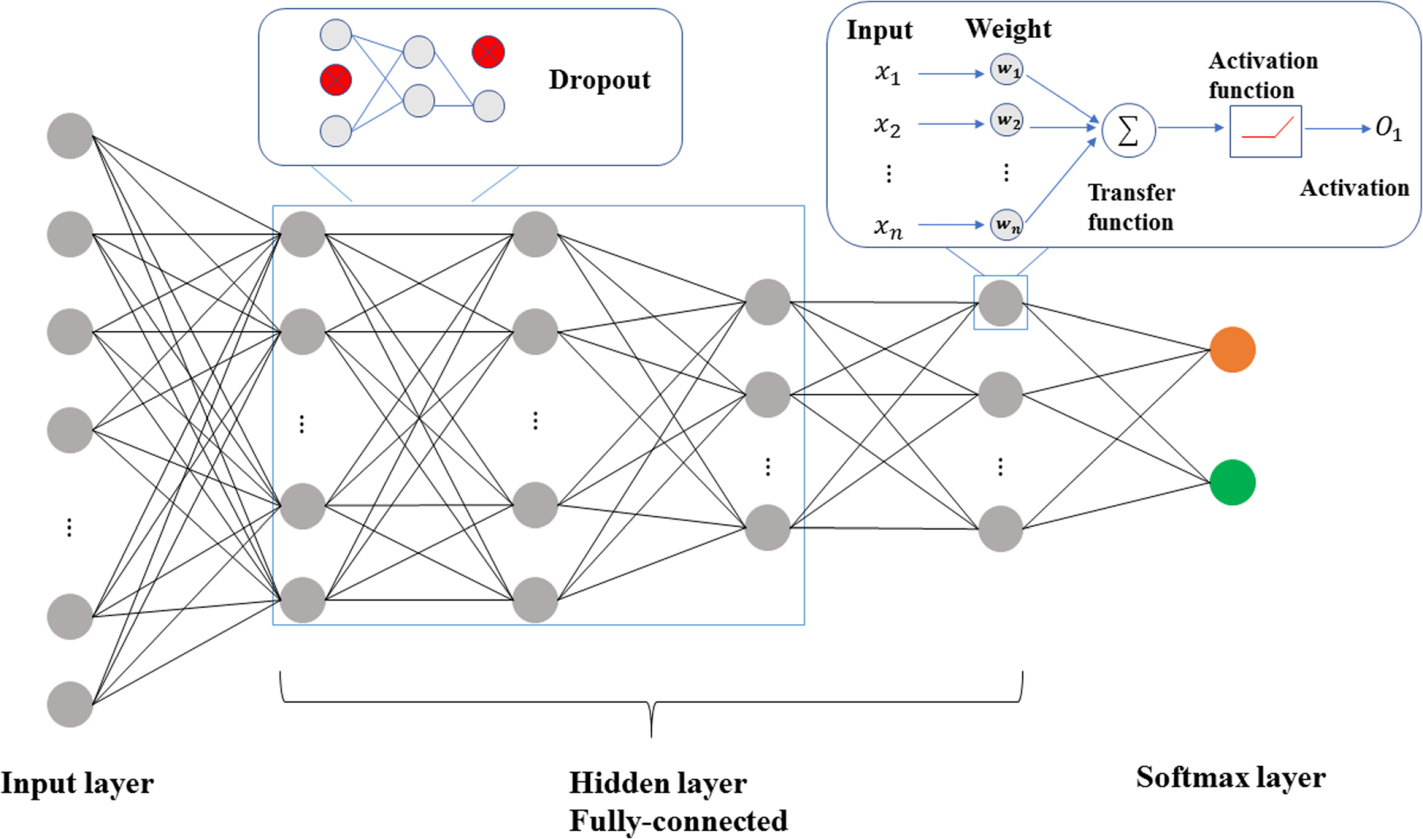
The framework of the neural network. A total of six neural levels were implemented. To reduce overfitting, we used the dropout method in every layer except the last one. Additionally, the previous layers used the RELU function to avoid gradient diffusion. We introduced the softmax function to classify the last layer

Deep learning has been focused in recent years in the AI field, and multilayer perceptron (MLP) is one of these deep learning frameworks. We constructed a six-layer MLP (including input and output layers), which is shown in Fig. 7. The first layer of the network is the input layer, which is used to input data. The number of neurons in the first layer is equal to the feature’s dimensions for the input data. The activation function is used to activate neurons and transfer data to the next layer.

During the neural network training process, we used a Rectified Linear Unit (ReLU) as the activation function [42], and a softmax loss function [43] in our model. Additionally, the error backpropagation algorithm [44] and the mini-batch gradient descent algorithm were utilized to optimize the parameters. In the transmission of data from input to output, neural networks could learn and extract underlying features of the data. The last layer was the output layer, and the number of neurons in this layer denoted the number of categories. We adopted the softmax function [43], which is commonly used in classification as an activation function in the output layer. The mini-batch gradient descent algorithm was meant to use a small part of the training samples to train the model each time, which could reduce the calculation of the gradient descent method. The optimal value for batch size was 40. To accelerate the rate of gradient descent and suppress the oscillation, we adopted a momentum item in the process of optimizing weights and bias. To reduce overfitting, we used dropout methods in every layer of the neural network except for the last layer. This way, not every neuron had a full connection, which could reduce overfitting and speed up the training of the neural network. Detailed parameter information about the neural network is shown in Additional file 7: Table S7. The predictor for the above deep learning framework is called DeepAcet.

### Measurements of performance

The common performance measures of accuracy (Acc), specificity (Sp), sensitivity (Sn), Receiver Operating Characteristic (ROC) curves, Area Under the ROC curve (AUC) and Matthews correlation coefficient (MCC) were used to assess the performance of the predictor. Accuracy indicates the percentage of the test set correctly predicted. The specificity (also called the true negative rate) represents the proportion of negatives that are correctly predicted. The sensitivity (also called the true positive rate or the recall) measures the proportion of positives that are correctly predicted. The MCC accounts for the true and false positives as well as negatives, and is usually regarded as a balanced measure [24]. Importantly, 4-, 6-, 8-, and 10-fold cross-validation were performed. The common measurements are found below

2 $$ \left\{\begin{array}{c}\begin{array}{c}\mathrm{Sp}=\frac{TN}{TN+ FP}\\ {}\mathrm{Sn}=\frac{TP}{FN+ TP}\end{array}\ \\ {}\begin{array}{c}\mathrm{Acc}=\frac{TP+ TN}{TP+ TN+ FP+ FN}\ \\ {}\mathrm{MCC}=\frac{TP\times TN- FP\times FN}{\sqrt{\left( TP+ FN\right)\left( TN+ FP\right)\left( TP+ FP\right)\left( TN+ FN\right)}}\end{array}\end{array}\ \right. $$

## Additional files

Additional file 1: **Table S1.** The performance of six combined features without F-score. The table shows the performance measures (Accuracy, Specificity, Sensitivity, AUC, MCC) for the combination of six encoding methods. (XLSX 11 kb)

Additional file 2: **Table S2.** The F-score values of each feature. The table shows the F-score values of the 3972 features obtained by six encoding methods. (XLSX 100 kb)

Additional file 3: **Table S3.** – The performance of different lengths of input peptides. The table shows the performance measures (Accuracy, Specificity, Sensitivity, AUC, MCC) for different lengths (21, 23, 25, 27, 29, 31, 33, 35) of fragments. (XLSX 12 kb)

Additional file 4: **Table S4.** The training set for lysine acetylation. The table shows all training sets (positive and negative fragments). (XLSX 1137 kb)

Additional file 5: **Table S5**. - The independent test set for lysine acetylation. The table shows all independent test sets (positive and negative fragments). (XLSX 314 kb)

Additional file 6: **S6.** Six encoding feature constructions. The supplementary material describes six encoding schemes. (DOCX 20 kb)

Additional file 7: **Table 7.** Detailed parameter information about the neural network. The table contains the parameter information of MLP: the number of neurons in each layer, activation function, momentum, loss function, batch size, and learning rate. (XLSX 16 kb)
