# Supplementary material for: A deep learning method to more accurately recall known lysine acetylation sites
Source: BMC Bioinformatics. 2019 Jan 23;20:49. doi: 10.1186/s12859-019-2632-9 (PMC6343287; doi:10.1186/s12859-019-2632-9)
Supplement: Supplementary file 6 — S6. Six encoding feature constructions. The supplementary material describes six encoding schemes. (DOCX 20 kb) [file 12859_2019_2632_MOESM6_ESM.docx]

**Six encoding feature constructions**

**One-hot encoding**

The amino acids within a small range around the acetylation site are primary sequence features and have proven to be useful information for lysine acetylation sites prediction in previous studies [1]. These features can be used to represent protein sequences. We adopted one-hot encoding scheme to transform protein fragments into numeric vectors. Thus, each of the 21 different amino acids (20 amino acid plus the gap-filling residue “X”) was encoded into a 21-dimensional vector that contained only 0 and 1. We arranged the amino acids in the order of ARNDCQEGHILKMFPSTWYVX. Amino acid A was represented as (1,0,0,0,0,0,0,0,0,0,0,0,0,0,0,0,0,0,0,0,0), R was represented as (0,1,0,0,0,0,0,0,0,0,0,0,0,0,0,0,0,0,0,0,0), and so on. Therefore, the dimension of the numeric vector is 21*31.

**BLOSUM62 matrix**

BLOSUM (Blocks Substitution Ma) matrices have belonged to the most common substitution matrix series for protein homology search and sequence alignments since their publication in 1992 [2]. Essential characters of protein evolution can be learned from analysis of aligned protein sequences [3]. So, BLOSUM62 matrix can be used to encode protein sequences, which contain the evolutionary information of sequences. In this work, we assigned zero the gap-filling residue “X”. Each row of the matrix represents one amino acid, for example, the first row of the matrix represents amino acid A. In this way, the dimension of the numeric vector is 21*31.

**Composition of *K*-space amino acid pairs (CKSAAP)**

The CKSAAP encoding scheme reflects the information of amino acid pairs in small range within the peptides. There are 441 different amino acid pairs for the 21 amino acids. A sliding window with *K*-space (amino acid pair separated by *K* amino acid) was used to scan through the fragments and count all amino acid pairs (e.g., AXXV is a two-space amino acid pair and *K*=2). We defined an amino acid pair as $A_{i}A_{j}(i,j=1,2,\cdots,21)$.The frequency $f_{i,j}$ of all possible amino acid pairs is defined as

$$f_{i,j}=\frac{{Num(A}_{i}A_{j})}{L-K-1} (i,j=1,2,\cdots,21) (1)$$

where *L* represents the length of fragments. After calculating the frequency of all possible amino acid pairs, we can get a 441-dimensional vector ${(f_{1,1},f_{1,2},\cdots,f_{1,21},f_{2,1},f_{2,2}\cdots f_{2,21},\cdots{,f}_{21,1},f_{21,2},\cdots f_{21,21})}_{441}$. In order to get more information about fragment dependency, we took *K* from 0 to 4 and obtained a 2205-dimensional feature vector.

**Information gain (IG)**

Shannon Entropy was defined as a unique function that represents the average amount of information for a set of objects according to their probabilities [4]. It can be used to measure the conservation of amino acids in fragments. We used window-wise entropy that is calculated by probability of the individual amino acid to generate one numeric feature [5]. For a given fragment, it can be calculated as

$$H=-\sum_{i=1}^{21} p_{i}\log\left( p_{i} \right) (2)$$

where$p_{i}$ represents the probability of an amino acid (20 amino acid plus the gap-filling residue “X”) in the fragment. The probability $p_{i}$ is equal to the total number of amino acid $i$ in the fragment divided by the length of the fragment. When the amino acids in the fragment are the same, Shannon entropy takes 0. Relative Entropy is also known as Kullback-Leibler distance and defined as

$$RE=\sum_{i=1}^{21} p_{i}log(\frac{p_{i}}{p_{0}}) (3)$$

where $p_{0}=\frac{1}{L}$, $L$represents the length of the fragment. Information Gain measures the information transformation from the background or random state to the state influenced by the class no matter positive or negative class [5]. It is derived from the following formula

$$IG=H-RE. (4)$$

Finally, a fragment can be represented by the IG value.

**Physicochemical and biochemical properties**

AAindex is a database of numerical indices representing various physicochemical and biochemical properties of amino acids [6]. There are 566 entries in Amino Acid Index Database (<http://www.genome.jp/dbget-bin/www_bfind?aaindex>). We chose the common 14 physicochemical properties from Amino Acid Index Database and normalized them. The details of the 14 physicochemical properties were Hydrophobicity, Polarity, Polarizability parameter, Transfer energy, Organic solvent/water, Accessible surface area, Net charge, Molecular weight, pK-N, pK-C, Melting point, Optical rotation, Entropy of formation, Heat capacity, and Absolute entropy [6]. For a given fragment, each amino acid can be represented by 14 values. Thus, a fragment is encoded into a 434-dimensional vector.

**Position-specific scoring matrix (PSSM)**

To get information about the sequential evolution, we can exploit the data of the position-specific scoring matrix. Position-specific scoring matrix (PSSM) is constructed according to the model proposed by Vacic, Iakoucheva and Radivojac [7], PSSM [8]. Let *P* and *N* represent the flaking regions of positive and negative sample, $N^{+}$ and $N^{-}$represent the number of fragments in the positive and negative dataset, respectively. $P_{i}$ is the $i-$th fragment in the positive dataset, $P_{ij}$ is the $j-$th position in the $i-$th fragment. A binary vector $X_{P}^{j,a}$ can be obtained for each symbol $a$ from the 21 amino acids

$$X_{P}^{j,a}=\left( I_{1},I_{2},\cdots,I_{N^{+}} \right) \left( 5 \right)$$

where $I_{i}$ can be calculated using the following formula

$I_{i}=\left\{ \begin{aligned} \begin{matrix} 1 & P_{ij}=a \end{matrix} \\ \begin{matrix} 0 & P_{ij}=a \end{matrix} \end{aligned} \right. . (6)$

The vector $X_{N}^{j,a}$ for negative samples can be formed in the same way. For each set of $X_{P}^{j,a}$ and $X_{N}^{j,a}$, a *p*-value was obtained via two-sample t-test [7]. Then, we constructed the following matrix $V_{PSSM}$

$$V_{PSSM}=\left[ \begin{matrix} \begin{matrix} V_{1,1} & V_{1,2} \\ V_{2,1} & V_{2,2} \end{matrix} & \begin{matrix} \cdots& V_{1,L} \\ \cdots& V_{2,L} \end{matrix} \\ \begin{matrix} \vdots& \vdots\\ V_{21,1} & V_{21,2} \end{matrix} & \begin{matrix} \vdots& \vdots\\ \cdots& V_{21,L} \end{matrix} \end{matrix} \right] . (7)$$

In this matrix, *L* is the length of the fragment.$V_{i,j}$ is the p-value of the $i-$th amino acid in the $j-$th position for a given positive and negative dataset. By calculating the frequency of each amino acid in each position of the fragments in positive dataset, we constructed the following matrix $F^{P}$

$$F^{P}=\left[ \begin{matrix} \begin{matrix} F_{1,1}^{P} & F_{1,2}^{P} \\ F_{2,1}^{P} & F_{2,2}^{P} \end{matrix} & \begin{matrix} \cdots& F_{1,L}^{P} \\ \cdots& F_{2,L}^{P} \end{matrix} \\ \begin{matrix} \vdots& \vdots\\ F_{21,1}^{P} & F_{21,2}^{P} \end{matrix} & \begin{matrix} \vdots& \vdots\\ \cdots& F_{21,L}^{P} \end{matrix} \end{matrix} \right] (8)$$

where $F_{i,j}^{P}$represents the frequency of the $i-$th amino acid in the $j-$th position. $F^{N}$can be constructed in the same way. Finally, we computed the following PSSM matrix

$$E_{PSSM}=\left[ \begin{matrix} \begin{matrix} E_{1,1} & E_{1,2} \\ E_{2,1} & E_{2,2} \end{matrix} & \begin{matrix} \cdots& E_{1,L} \\ \cdots& E_{2,L} \end{matrix} \\ \begin{matrix} \vdots& \vdots\\ E_{21,1} & E_{21,2} \end{matrix} & \begin{matrix} \vdots& \vdots\\ \cdots& E_{21,L} \end{matrix} \end{matrix} \right] (9)$$

where $E_{i,j}$ can be calculated

$$E_{i,j}=\left\{ \begin{matrix} \ln\left( \left| \delta_{i,j} \right|+1 \right) & \delta_{i,j}\geq0 \\ -\ln\left( \left| \delta_{i,j} \right|+1 \right) & \delta_{i,j}<0 \end{matrix} \right. (10)$$

$$\delta_{i,j}=\frac{F_{i,j}^{P}-F_{i,j}^{N}}{V_{i,j}} . (11)$$

If $E_{i,j}$>0, the $i-$th amino acid in the $j-$th position has a greater probability appearing in the positive fragment. Otherwise, it is more likely to be in the negative fragment.

References

1. Li T, Du P, Xu N: **Identifying human kinase-specific protein phosphorylation sites by integrating heterogeneous information from various sources**. *PLoS One* 2010, **5**(11):e15411.

2. Hess M, Keul F, Goesele M, Hamacher K: **Addressing inaccuracies in BLOSUM computation improves homology search performance**. *BMC Bioinformatics* 2016, **17**:189.

3. Zhang L-M, Liu X: **Significant residue features revealed by eigenvalue decomposition analysis of BLOSUM matrices**. *Physics Letters A* 2008, **372**(13):2282-2285.

4. Shannon CE: **A mathematical theory of communications**. *Bell System Technical Journal* 1948, **27**:379-423.

5. Ismail HD, Jones A, Kim JH, Newman RH, Kc DB: **RF-Phos: A Novel General Phosphorylation Site Prediction Tool Based on Random Forest**. *Biomed Res Int* 2016, **2016**:3281590.

6. Kawashima S, Pokarowski P, Pokarowska M, Kolinski A, Katayama T, Kanehisa M: **AAindex: amino acid index database, progress report 2008**. *Nucleic Acids Res* 2008, **36**(Database issue):D202-205.

7. Vacic V, Iakoucheva LM, Radivojac P: **Two Sample Logo: a graphical representation of the differences between two sets of sequence alignments**. *Bioinformatics* 2006, **22**(12):1536-1537.

8. Xie Y, Luo X, Li Y, Chen L, Ma W, Huang J, Cui J, Zhao Y, Xue Y, Zuo Z *et al*: **DeepNitro: Prediction of Protein Nitration and Nitrosylation Sites by Deep Learning**. *Genomics Proteomics Bioinformatics* 2018.
